# Endophytic fungal community structure and diversity in paphiopedilum barbigerum across different habitats

**DOI:** 10.1080/15592324.2026.2689805

**Published:** 2026-06-18

**Authors:** Yu Wu, Ping Yu, Fan Tian, Lang Huang, Shan Du, Cheng jiang Tan, Yuan mei Jia

**Affiliations:** a Forestry College, Guizhou University, Guiyang, Guizhou, People's Republic of China; b Guizhou Academy of Forestry / Key Laboratory of National Forestry and Grassland Administration on Biodiversity Conservation in Karst Mountainous Areas of Southwestern China, Guizhou, Guizhou, People's Republic of China; c Guizhou Maolan National Nature Reserve Administration, Libo, Guizhou, People's Republic of China

**Keywords:** *Paphiopedilum barbigerum*, endophytic fungi, community, diversity

## Abstract

**Objective:**

This study aimed to investigate the response patterns of root-associated fungal communities in the rare and endangered species *Paphiopedilum barbigerum* across different habitat types, and to identify key taxonomic groups that differentiate natural habitats from reintroduction sites, thereby providing a theoretical basis for the species artificial propagation and field reintroduction.

**Methods:**

Using PacBio high-throughput sequencing, we sequenced the ITS region of 21 root samples of *P. barbigerum* collected from seven sites representing three habitat categories: natural habitats within a nature reserve (SL, DE, YL, KL), natural habitats outside the reserve (FQ, KY), and a field reintroduction site (HG). Alpha diversity, beta diversity, LEfSe analysis, and co-occurrence network analysis were employed to characterize root-associated fungal community structure, diversity, and habitat-driven assembly patterns.

**Results:**

A total of 792,178 clean reads were obtained and classified into 8 phyla, 37 classes, 134 orders, 352 families, 1,012 genera, and 2,311 species. The communities were dominated by Ascomycota (52.90%–91.41%) and Basidiomycota (7.79%–54.33%). Key findings include: (1) Fungal species richness was significantly higher in natural habitats within the reserve than at the reintroduction site and natural habitats outside the reserve, with specific enrichment of desiccation-tolerant taxa such as Chaetothyriales and *Exophiala* (relative abundance 12.58%–33.53%). (2) The fungal community structure at the reintroduction site converged with that of natural habitats outside the reserve. Core OTUs included *Epulorhiza* sp., *Cladosporium* sp., and *Lachancea thermotolerans*, all of which exhibited significantly higher relative abundances in these two habitat types; notably, *Epulorhiza* sp. reached a relative abundance of 44.79% at the reintroduction site, suggesting a key role in host growth and development.

**Conclusion:**

Habitat heterogeneity is associated with adaptive restructuring of the root-associated fungal community by selecting for distinct functional guilds: desiccation-tolerant taxa are enriched in natural habitats within the reserve, whereas growth-promoting taxa are enriched in disturbed habitats. We recommend prioritizing the conservation of natural habitats within the reserve to preserve high fungal diversity and stress-tolerant guilds, and emphasize the targeted utilization and monitoring of core growth-promoting fungi such as *Epulorhiza* during reintroduction programs.

## 1. Introduction

*Paphiopedilum barbigerum* (Orchidaceae, *Paphiopedilum* Pfitz.) is a species of high ornamental value and considerable ecological significance. It is currently listed as Endangered (EN) on the IUCN Red List, included in Appendix I of CITES,^1^ and designated as a Class I National Key Protected Wild Plant in China—placing it among the highest-priority taxa for conservation. This species predominantly grows as an epiphyte on limestone scrub or within rock crevices in the karst landscapes of southwestern China,^2^ a harsh habitat characterized by persistent water and nutrient stress. Furthermore, its reliance on deceptive pollination results in low natural fruit set,^3^ and critical life-history stages depend heavily on symbiotic associations with specific mycorrhizal fungi.^4^ ^,^ ^5^ Habitat fragmentation and historical over-collection^6^ have further exacerbated wild population declines, pushing the species toward extreme endangerment. Concerted and targeted conservation action is therefore urgently needed.

Endophytes, primarily endophytic bacteria and fungi, co-evolve with their host plants and confer resistance to both biotic and abiotic stresses, generally without detrimental effects on the host.^7^ ^-^ ^9^ In orchids, the specialized mycorrhizal fungal symbiosis not only delivers key nutrients such as carbon and nitrogen to the host,^10^ but also enhances drought adaptation by improving water uptake and strengthening physiological and biochemical stress tolerance.^11^ Moreover, mycorrhizal fungi can reverse the protocorm developmental arrest caused by excessive auxin accumulation in *Paphiopedilum* species, promote differentiation, and reduce disease incidence through the secretion of antagonistic compounds.^12^ ^,^ ^13^ For instance, *Epulorhiza* sp. has been shown to induce lateral root formation and enhance root activity in *Dendrobium officinale* through symbiotic association, significantly promoting host growth.^14^ A significant positive correlation between fungal colonization and host stress-resistance phenotypes^15^ further suggests that fungal communities may mediate plant–environment interactions through metabolic exchange networks.

Next-generation sequencing, which targets hypervariable regions such as ITS through a culture-independent metagenomic strategy, now enables high-resolution characterization of endophytic fungal communities.^16^ Compared with earlier Sanger sequencing, high-throughput approaches offer greater depth and sensitivity, and are especially suitable for low-biomass samples such as fine orchid roots, permitting comprehensive profiling of root-associated fungal community structure and diversity without the need for cultivation.^17^ Leveraging this technology,^18^ demonstrated significant differences in fungal community structure among habitats of different *Paphiopedilum* species within subgenus *Brachypetalum*, with Basidiomycota and Ascomycota as the dominant phyla.^18^

For *P. barbigerum*, a karst endemic, existing studies have focused primarily on tissue culture and rapid propagation,^19^ population structure and demographic dynamics,^20^ and mycorrhizal symbiosis.^4^ Li ^21^ reported differences in mycorrhizal fungal diversity between native and ex situ conservation habitats using isolation, culture, and identification methods,^21^ However, systematic investigation into how root-associated fungal community structure differs across habitat types and how functional guilds undergo adaptive shifts in response to environmental heterogeneity remains lacking, We therefore propose that habitat heterogeneity drives the divergent assembly of root-associated fungal communities, and that functional groups exhibit adaptive transitions in response to water or nutrient stress. Within *Paphiopedilum*, environmentally driven effects are especially pronounced: recent work on the Critically Endangered (CR) congener *P. micranthum* revealed marked divergence in rhizosphere microbial community structure and soil properties between lithophytic and terrestrial ecotypes^22^; climatic and edaphic factors not only govern fungal community assembly in its habitats,^18^ but may also influence gene flow and adaptive evolution.^23^ To address this knowledge gap, we collected *P. barbigerum* samples from seven sites across three habitat categories—natural habitats within a nature reserve, a field reintroduction site, and natural habitats outside the reserve—and performed amplicon sequencing on the PacBio platform. We assessed community richness and evenness using Alpha diversity indices (ACE, Chao1, Shannon) and resolved habitat-driven community differentiation through Beta diversity analyses (PCoA, NMDS). Our objectives were to elucidate the impact of habitat variation on root-associated fungal community structure and diversity, to characterize the distribution patterns and ecological functions of core fungal taxa across habitats, and ultimately to provide a theoretical framework for artificial propagation, field reintroduction, and population recovery of *Paphiopedilum* species.

## 2. Materials and methods

### 2.1. Sample collection

Root samples of *Paphiopedilum barbigerum* were collected from seven sites across four regions in Guizhou Province, China: Maolan National Nature Reserve (hereafter Maolan Reserve), Fuquan City, Kaiyang County, and the field reintroduction base at Yunguanshan State-owned Forest Farm. The seven sites comprised four natural habitats within Maolan Reserve—Yaolan (YL), Dong'en (DE), Kenli (KL), and Shishang Forest at Kenli (SSSL, hereafter SL)—two natural habitats outside the reserve (Fuquan, FQ; Kaiyang, KY), and the reintroduction site at Yunguanshan (HG). At each site, 2–3-year-old roots were sampled from three healthy individuals spaced at least 2 m apart. All samples were immediately placed in sterile ziplock bags and preserved on dry ice.

### 2.2. Sample processing

Upon arrival at the laboratory, root samples were transferred individually into 50 mL sterile tubes containing phosphate-buffered saline (PBS). Roots were gently agitated with sterile forceps for 5 min to remove surface debris, then sequentially rinsed with sterile distilled water for 30 s, surface-sterilized in 70% ethanol for 2 min and 2.5% sodium hypochlorite for 5 min, and finally rinsed five times with sterile distilled water. After blotting dry with sterile filter paper, samples were stored on dry ice pending DNA extraction.

### 2.3. DNA extraction and high-throughput sequencing

Surface-sterilized root samples were ground in liquid nitrogen, and total genomic DNA was extracted using the E.Z.N.A. Plant DNA Kit (Omega Bio-tek, Norcross, GA, USA) according to the manufacturer's instructions. DNA integrity and purity were assessed by 1% agarose gel electrophoresis. The fungal internal transcribed spacer (ITS) region was amplified using the universal primer pair ITS1F (5′-CTTGGTCATTTAGAGGAAGTAA-3′) and ITS4R (5′-TCCTCCGCTTATTGATATGC-3′). Each 20 μL PCR reaction contained 4 μL of 5 × FastPfu Buffer, 2 μL of dNTPs (2.5 mmol/L), 0.8 μL of each primer (5 μmol/L), 0.4 μL of FastPfu Polymerase, 10 ng of template DNA, and ddH₂O to volume. The thermal cycling program was: 95 °C for 5 min; 25 cycles of 95 °C for 30 s, 58 °C for 30 s, and 72 °C for 45 s; followed by a final extension at 72 °C for 10 min. PCR products were quantified using QuantiFluor™-ST and subjected to high-throughput sequencing on the PacBio platform by Shanghai Ling'en Biotechnology Co., Ltd.

### 2.4. Sequence processing and OTU clustering

Raw sequencing data were processed with SMRT Link software (v11.0) to generate demultiplexed circular consensus sequences (CCS), with a minimum pass number of 3 and a minimum predicted accuracy of 0.99. Raw reads were filtered on the SMRT Portal platform to retain high-quality sequences 1000–1800 bp in length. Barcodes and primers were removed using the lima workflow (Pacific Biosciences, https://lima.how/). High-quality sequences were clustered into operational taxonomic units (OTUs) at 98.65% similarity using UPARSE (version 10, http://drive5.com/uparse).^24^ OTU sequences were taxonomically annotated using the uclust algorithm^25^ (https://github.com/topics/uclust) against the Unite fungal database^26^ (Release 8.2, http://unite.ut.ee/index.php) at a confidence threshold of 80%.

### 2.5. Diversity and statistical analyses

Alpha and beta diversity analyses were performed on the Ling'en Biotechnology Cloud Platform (http://www.cloud.biomicroclass.com/CloudPlatform/home). Following rarefaction, Alpha diversity indices—including Coverage, Chao1, ACE, Shannon, and Simpson—were calculated to assess sequencing depth, species richness, and community diversity. Principal component analysis (PCA)^27^ and principal coordinate analysis (PCoA)^28^ were used for dimensionality reduction and visualization of community structure. Non-metric multidimensional scaling (NMDS) based on Bray–Curtis distances was applied to verify ordination robustness, and hierarchical clustering (UPGMA) was performed to illustrate similarity relationships among samples. To quantify the contribution of habitat grouping to community variation and assess statistical significance, permutational multivariate analysis of variance (PERMANOVA, also termed Adonis) was conducted based on the Bray–Curtis distance matrix with 999 permutations; R² values and *P* values were calculated for each grouping factor.

### 2.6. Differential taxa and biomarker identification

Linear discriminant analysis effect size (LEfSe) was employed for multi-group comparison. The Kruskal–Wallis rank sum test was first used to identify taxa with significant differential abundance among groups, followed by linear discriminant analysis (LDA) to estimate the effect size of each differentially abundant taxon (biomarker).^29^ The default significance threshold was set at *P* < 0.05, and the LDA score threshold at 2.0.

### 2.7. Co-occurrence network analysis

A co-occurrence network was constructed based on the fungal ITS sequencing dataset. Spearman's rank correlation coefficients were calculated between all OTU pairs; only correlations with |r| > 0.8 and *P* < 0.05 were retained. OTUs with a relative abundance >0.01% and a detection rate > 60% across samples were included. In the resulting network, nodes represent OTUs and edges represent significant correlations. Network modeling was performed using the WGCNA package in R. Keystone taxa were identified through Zi-Pi analysis,^30^ ^,^ ^31^ where nodes were classified as: peripherals (Zi < 2.5, Pi < 0.62), module hubs (Zi > 2.5, Pi < 0.62), connectors (Zi < 2.5, Pi > 0.62), and network hubs (Zi > 2.5, Pi > 0.62).

## 3. Results and analysis

### 3.1. Sequencing output of root-associated endophytic fungi across habitats

**Figure 1.**
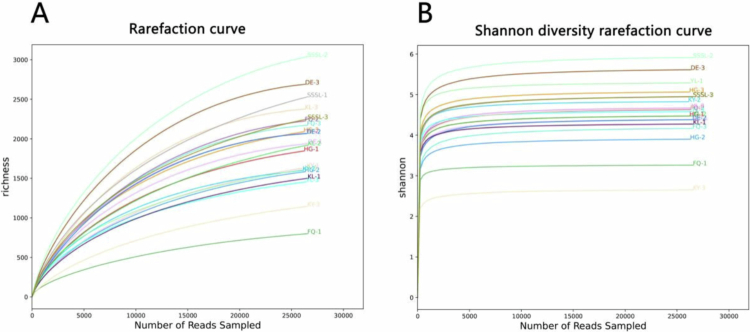
Dilution curves (A) and Shannon-Wiener curves (B) of root samples from *Paphiopedilum barbigerum* in different habitats. Note: Yaolan Sample Plot, Guizhou Maolan Reserve (Sample YL), Dong'en Sample Plot, Guizhou Maolan Reserve (Sample DE), Kenli Sample Plot, Guizhou Maolan Reserve (Sample KL), Epilithic Forest in Kenli Sample Plot, Guizhou Maolan Reserve (Sample SL), Fuquan City Sample Plot (Sample FQ), Kaiyang County Sample Plot (Sample KY), Wild Reintroduction Base at Yunguan Mountain State-Owned Forest Farm, Guizhou Province (Sample HG) .

High-throughput sequencing of root samples from six natural habitats and one field reintroduction site yielded a total of 792,178 clean reads (507,095,367 bp) across 21 samples, with sequence lengths primarily ranging from 597 to 760 bp. Rarefaction curves for all samples approached saturation as sequencing depth increased, indicating that the sequencing effort adequately captured the majority of fungal species present (Figure 1A). Shannon–Wiener curves similarly plateaued with increasing sequencing depth, confirming that the data volume was sufficient to robustly represent the taxonomic composition and structure of the fungal communities in each sample (Figure 1B).

### 3.2. Alpha diversity of root-associated endophytic fungi across habitats

**Figure 2.**
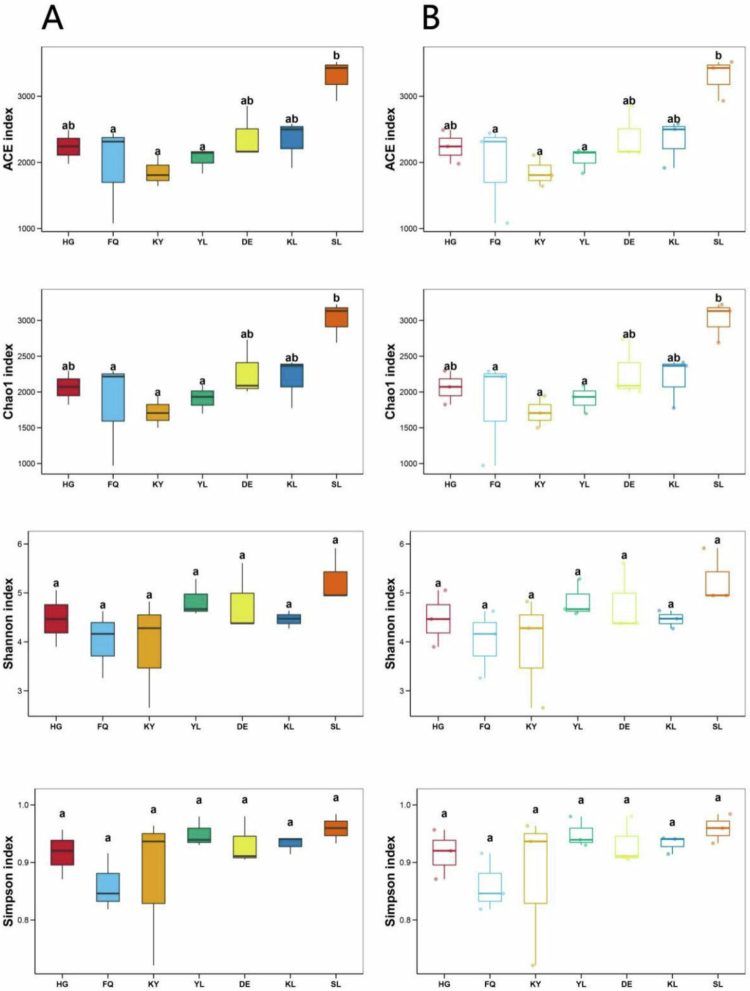
Comparative Analysis of Alpha Diversity Indices and Intergroup Differences in Endophytic Fungal Communities of *Paphiopedilum barbigerum* Roots across Different Habitats. Note: Panel A displays the distribution of Alpha diversity indices for in-situ sample sites (including Shishang Forest SL, Dong'en DE, Yaolan YL, Kaiyang KL, etc.); Panel B shows the distribution of Alpha diversity indices for artificial/disturbed sample sites (including Huigui HG, Fuquan FQ, Kaiyang KY, etc.). From top to bottom, the figure illustrates the ACE index, Chao1 index, Shannon index, and Simpson index. The boxplots represent the median (horizontal line), interquartile range (box), and maximum/minimum values (whiskers) for each index. Different lowercase letters indicate statistically significant differences according to Tukey’s HSD test (*p* < 0.05), where identical letters denote no significant difference and distinct letters denote significant differences.

Alpha diversity metrics were calculated to assess the richness and evenness of endophytic fungal communities in *P. barbigerum* roots across habitats (Figure 2; Table 1). Overall, species richness differed markedly among sites, whereas community evenness remained relatively stable. Specifically, Chao1 and ACE indices revealed that species richness at SL was significantly higher than at HG, KY, and several other sites (*P* < 0.05) (Figure 2). In contrast, Shannon and Simpson indices, which reflect community evenness and structure, showed no significant differences among sites (*P* > 0.05). Numerically, the highest richness values were recorded at SL and DE, while the highest diversity indices were observed at SL and YL; richness indices at FQ and KY were consistently low (Table 1). These results suggest that habitat variation primarily affects the number of endophytic fungal species without substantially altering the evenness of community composition.

**Table 1.** Alpha diversity analysis of root endophytic fungi in *Paphiopedilum barbigerum* across different habitats.

Sample	Sobs	Chao1	ACE	Shannon	Simpson	Pielou	Faith-PD
HG	1834.67	2063.62	2236.67	4.48	0.92	0.59	2.92
FQ	1731.33	1825.47	1944.93	4.02	0.85	0.54	3.53
KY	1459.33	1717.18	1854.42	3.92	0.87	0.54	0.79
YL	1711.67	1909.32	2052.96	4.85	0.95	0.65	2.09
DE	2228.67	2289.20	2390.74	4.79	0.93	0.62	2.88
KL	1932.00	2504.45	2333.82	4.46	0.93	0.59	2.51
SL	2605.00	3014.69	3291.96	5.27	0.96	0.67	2.49

Note: Values represent the mean of three replicates per site. Sobs, observed species richness; Faith_PD, Faith's phylogenetic diversity.

### 3.3. Community composition of root-associated endophytic fungi across habitats

**Figure 3.**
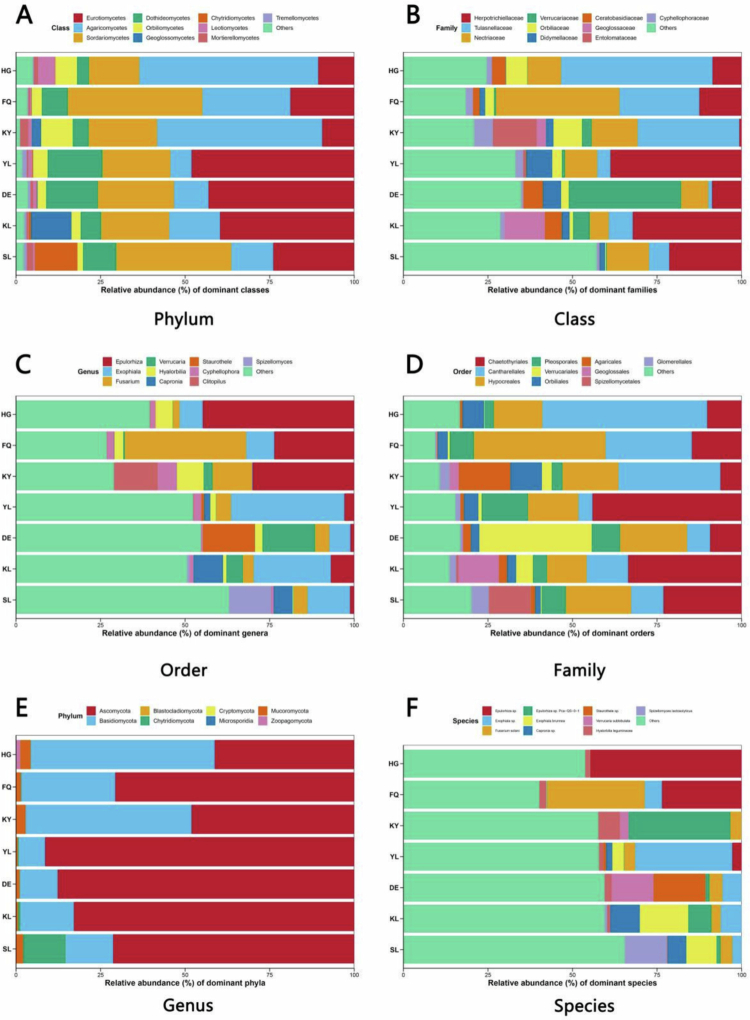
Distribution maps of root endophytic fungal taxa across taxonomic hierarchies in *Paphiopedilum barbigerum* from different habitats. Note: The Y-axis indicates the sample names; the X-axis shows the percentage of relative abundance.

Taxonomic classification of root-associated endophytic fungi across the seven sites identified 8 phyla, 36 classes, 133 orders, 351 families, 1,012 genera, and 2,310 species, excluding unclassified fungi. Species numbers varied substantially among sites (Table 2), ranking from highest to lowest as follows: SL (1,012 species) > DE (917) > KL (688) > YL (662) > KY (570) > FQ (500) > HG (422), indicating a marked effect of habitat type on fungal species richness. Dominant taxa also differed sharply among habitat categories (Table 2, Figure 3). The reintroduction site (HG) was dominated by Basidiomycota (54.33%) and Tulasnellaceae (44.79%). At FQ, a natural habitat outside the reserve, Ascomycota predominated (70.68%), with *Fusarium* accounting for 35.86% of the community. Within the reserve (YL, DE, KL, SL), Ascomycota was likewise dominant (71.32%–91.41%), and *Exophiala* consistently exhibited high relative abundance (12.58%–33.53%). These findings suggest that habitat category—natural habitats within the reserve, the reintroduction site, and natural habitats outside the reserve—is a major factor driving divergence in root-associated fungal community composition in *P. barbigerum*.

**Table 2.** Dominant fungal groups of root endophytic fungi in *Paphiopedilum barbigerum* across different habitats.

Site	Dominant phylum	Dominant class	Dominant order	Dominant family	Dominant genus	Dominant species
HG​	Ascomycota (52.90%) Basidiomycota (54.33%)	Agaricomycetes (14.00%) Sordariomycetes (14.86%) Eurotiomycetes (10.66%)	Cantharellales (48.73%) Hypocreales (14.30%) Chaetothyriales(10.21%)	Tulasnellaceae (44.79%) Unclassified (9.90%) Nectriaceae(9.85%)	*Epulorhiza* (44.79%) *Exophiala* (6.85%) *Hyalorbilia* (5.07%)	*Epulorhiza* sp. (44.79%) *Exophiala* sp. 050716 A (4.24%)
FQ	Ascomycota (70.68%) Basidiomycota (27.77%)	Agaricomycetes (26.02%) Sordariomycetes (39.76%) Eurotiomycetes (18.89%)	Hypocreales(38.90%) Cantharellales(25.53%) Chaetothyriales(14.72%)	Nectriaceae(36.48%) Tulasnellaceae(23.64%) Cucurbitariaceae (5.11%)	*Fusarium*(35.86%) *Epulorhiza* (23.64%) *Exophiala*(8.33%)	*Fusarium solani* (28.76%) *Epulorhiza* sp. (23.56%) *Exophiala* sp. 050716 A (5.12%)
KY	Ascomycota (48.12%) Basidiomycota (49.01%)	Agaricomycetes (48.83%) Sordariomycetes (20.19%) Eurotiomycetes (9.49%) Orbiliomycetes (9.32%)	Cantharellales(30.15%) Hypocreales(16.52%) Agaricales(15.22%)	Tulasnellaceae(30.09%) Nectriaceae(13.55%) Entolomataceae (12.95%) Orbiliaceae (8.40%)	*Epulorhiza*(30.08%) *Clitopilus* (12.94%) *Fusarium*(11.59%)	*Epulorhiza* sp. Pca-QS-0-1 (30.04%) *Clitopilus prunulus* (12.54%) *Hyalorbilia leguminacea* (6.37%)
YL	Ascomycota (91.41%) Basidiomycota (7.79%)	Eurotiomycetes (48.11%) Sordariomycetes (20.05%) Dothideomycetes(16.22%)	Chaetothyriales(44.13%) Hypocreales(14.86%) Pleosporales (13.73%)	Herpotrichiellaceae(38.80%) Nectriaceae(9.49%) Didymellaceae (7.68%)	*Exophiala* (33.53%) *Fusarium*(4.30%) *Paraboeremia* (4.20%)	*Exophiala* sp. (28.73%) *Paraboeremia putaminum* (4.16%) *Exophiala brunnea* (3.41%)
DE	Ascomycota (87.71%) Basidiomycota (11.12%)	Eurotiomycetes(43.10%) Sordariomycetes(22.46%) Dothideomycetes(15.33%)	Verrucariales (33.22%) Hypocreales(19.79%) Chaetothyriales(9.33%)	Verrucariaceae (33.22%) Herpotrichiellaceae(8.76%) Nectriaceae(8.01%)	*Verrucaria* (15.57%) *Staurothele* (15.35%) *Exophiala*(6.26%)	*Staurothele* sp. (15.35%) *Verrucaria sublobulata* (12.39%) *Exophiala* sp. (5.68%)
KL	Ascomycota (82.90%) Basidiomycota (15.77%)	Eurotiomycetes(39.69%) Sordariomycetes(20.08%) Agaricomycetes(15.05%)	Chaetothyriales(33.57%) Geoglossales (11.97%) Pleosporales(11.71%)	Herpotrichiellaceae(32.20%) Tulasnellaceae(7.12%) Nectriaceae(5.59%)	*Exophiala*(22.84%) *Trichoglossum*(11.96%) *Capronia* (8.73%)	*Exophiala brunnea* (14.31%) *Trichoglossum durandii* (11.96%) *Capronia* sp. (8.73%)
SL	Ascomycota (71.32%) Basidiomycota (13.99%)	Sordariomycetes(33.97%) Eurotiomycetes(23.96%) Chytridiomycetes(12.54%) Agaricomycetes(12.42%)	Chaetothyriales(23.13%) Hypocreales(19.29%) Spizellomycetales(12.43%)	Herpotrichiellaceae(21.42%) Nectriaceae(12.46%) Spizellomycetaceae(12.42%)	*Exophiala*(12.58%) *Spizellomyces* (12.43%) *Capronia*(5.59%)	*Spizellomyces lactosolyticus* (12.43%) *Exophiala brunnea* (8.92%) *Capronia* sp. (5.55%)

### 3.4. Community structure and composition of dominant taxa

**Figure 4.**
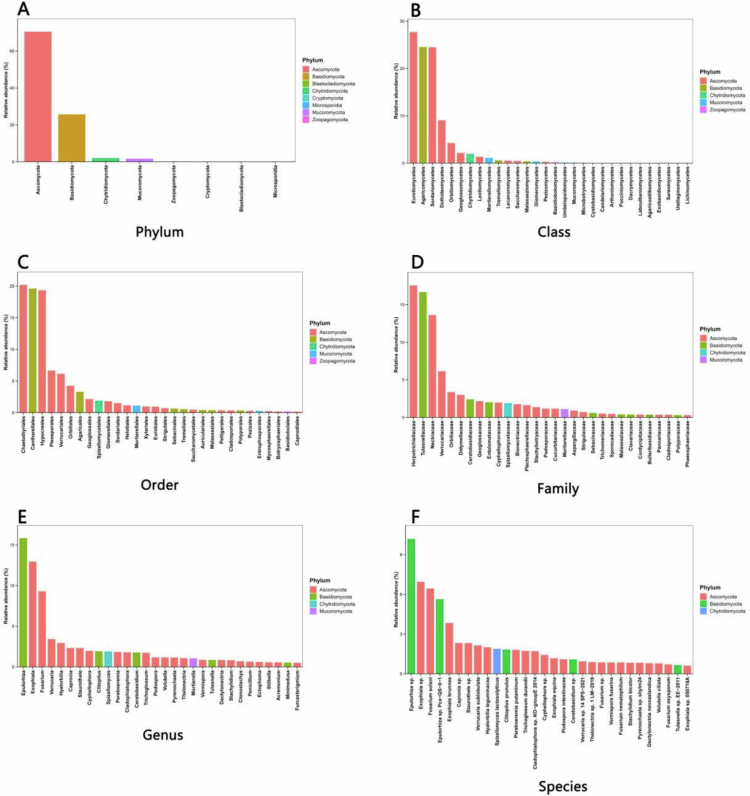
Bar chart of dominant species across taxonomic ranks. Note: A (phylum), B (class), C (order), D (family), E (genus), F (species) represent the stacked bar charts of relative abundance at different taxonomic levels. The color of the legend on the right represents the phylum-level classification units, and the lowercase letters at the top of the bars indicate the significant differences between groups (*P* < 0.05).

The dominant phyla in the root-associated endophytic fungal community of *P. barbigerum* were Ascomycota and Basidiomycota. At the class level, the community was dominated by Eurotiomycetes, Agaricomycetes, Sordariomycetes, and Dothideomycetes. The dominant orders were Chaetothyriales, Cantharellales, and Hypocreales. At the family level, Herpotrichiellaceae, Tulasnellaceae, Nectriaceae, and Verrucariaceae prevailed. The dominant genera included *Epulorhiza*, *Exophiala*, and *Fusarium*, among which the most abundant species were *Epulorhiza* sp., *Exophiala* sp., *Fusarium solani*, and *Epulorhiza* sp. Pca-QS-0-1 (Figure 4).

### 3.5. Core OTU analysis

**Figure 5.**
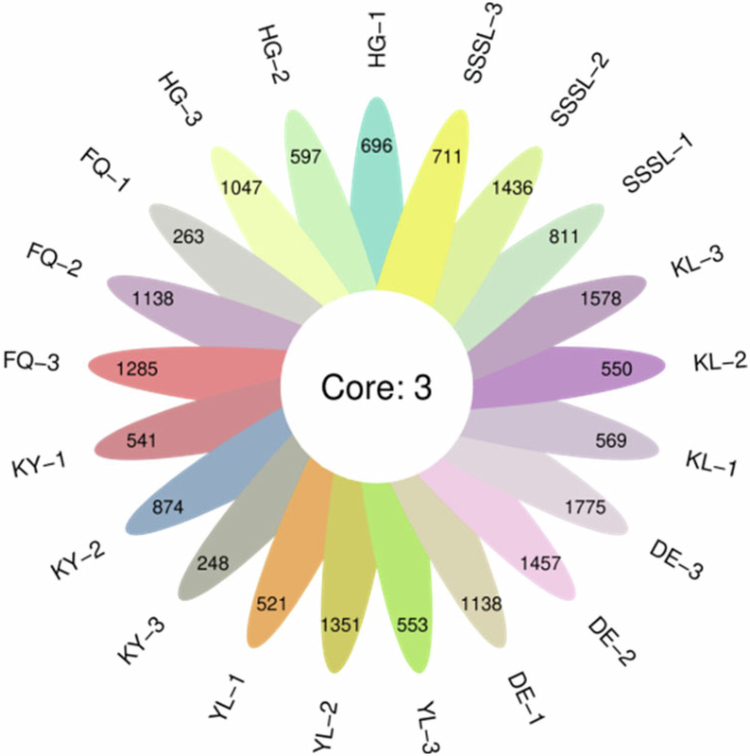
Core OTUs petal diagram. Note: The central ‘Core: 3’ indicates the number of core operational taxonomic units (OTUs) shared across all sampling sites. The radiating branches represent different sites ( HG, FQ, KY, YL, DE, KL, SL) , with the number on each branch corresponding to the total relative abundance (or read count) of core OTUs in that site, and colors differentiate the sites.

**Figure 6.**
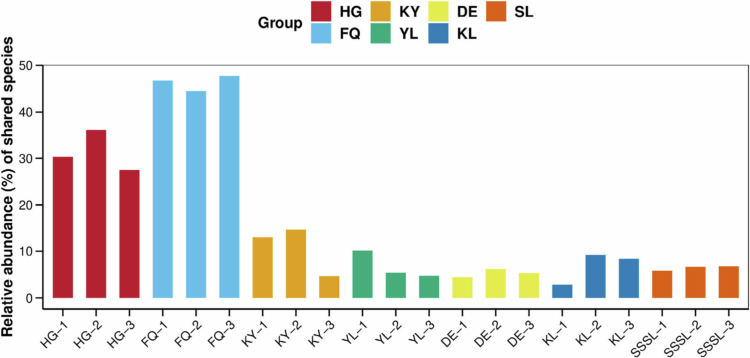
Bar chart of the total relative abundance of core OTUs across samples. Note: The bar height represents the total relative abundance of core OTUs in each sample, and the bar color indicates the sample group membership.

A flower plot was constructed to visualize OTU distribution across samples. Each colored ellipse represents one sample, with the number inside indicating OTUs unique to that sample. The number in the central white circle denotes core OTUs detected across all samples, which totaled three: OTU1, OTU65, and OTU453, taxonomically assigned as *Epulorhiza* sp., *Cladosporium* sp., and *Lachancea thermotolerans*, respectively. We further examined the taxonomic composition of these core OTUs and calculated their cumulative relative abundance in each sample (Figure 5). The highest cumulative relative abundance was recorded at FQ (46.30%), followed by HG (31.29%); values at the remaining five sites were substantially lower and ranked as KY (10.82%) > KL (6.80%) > YL (6.75%) > SL (6.41%) > DE (5.30%) (Figure 6).

### 3.6. Beta diversity of root-associated endophytic fungi across habitats

**Figure 7.**
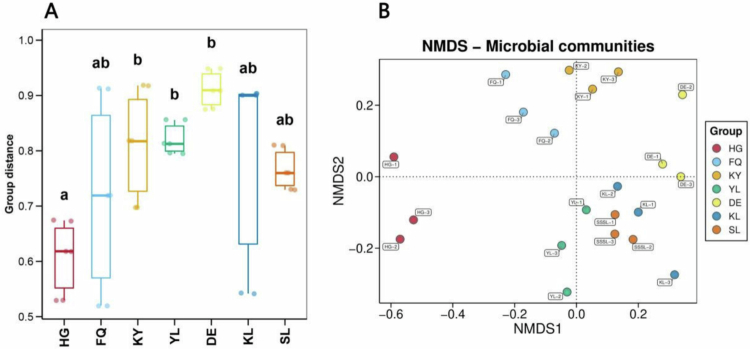
Box Plot and Heatmap of Bray-Curtis Distance for endophytic fungi communities in the roots of *Paphiopedilum barbigerum* from different habitats. Note: Figure A is a between-group distance boxplot (showing the number of core OTUs and intergroup differences), and Figure B is an NMDS ordination plot of community structure (colors differentiate sample sites).

**Figure 8.**
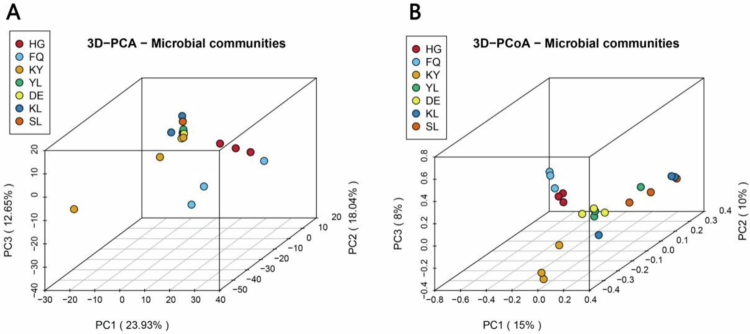
PCA and PCoA analysis of endophytic fungi communities in the roots of *Paphiopedilum barbigerum* from different habitats and wild reintroduction sites. Note: FigureA: 3D-PCA (variance explained by PC1/PC2/PC3: 23.93%/18.04%/12.65%); Figure B: 3D-PCoA (variance explained by PC1/PC2/PC3: 15%/9%/10%). Colors indicate sample sites (HG, FQ, KY, YL, DE, KL, SL), Point proximity reflects community similarity; conversely, points farther apart indicate greater dissimilarity.

The HG group exhibited the lowest median Bray–Curtis distance (approximately 0.62), which was significantly lower than that of DE, KY, and several other groups (*P* < 0.05), indicating the highest within-group compositional similarity and community stability. In contrast, the DE group showed the highest median distance (approximately 0.91), reflecting the greatest within-group variation (Figure 7A). Non-metric multidimensional scaling (NMDS) analysis (stress = 0.20) visually resolved the divergence trends in community structure (Figure 7B). Samples from the HG group clustered tightly on the left side of the ordination, demonstrating strong within-group aggregation; FQ samples grouped in the upper-left region, whereas DE samples were positioned on the right, distantly separated from HG. These patterns indicate that habitat type is a key driver of structural differentiation in the root-associated fungal community of *P. barbigerum*, with distinct habitats shaping markedly different fungal assemblages. Principal coordinate analysis (PCoA) and principal component analysis (PCA) based on Beta diversity further revealed that fungal communities at HG and FQ were compositionally similar, as were those at YL and DE, and at SL and KL (Figure 8A, 8B). To quantify the contribution of habitat type to community variation, permutational multivariate analysis of variance (PERMANOVA) based on Bray–Curtis distances was performed. The results confirmed that habitat type had a highly significant effect on root-associated endophytic fungal community structure (*R*² = 0.49967, *F* = 4.6143, *P* = 0.001).

### 3.7. Hierarchical clustering and community composition at the genus level

**Figure 9.**
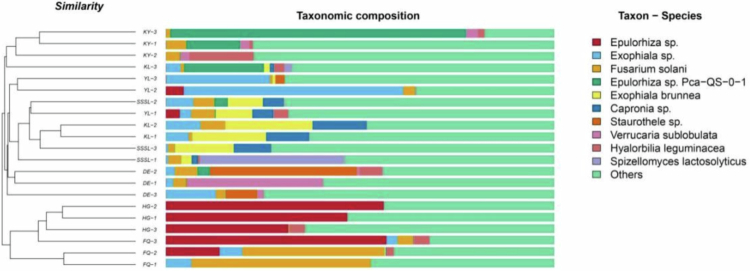
Cluster and columnar analysis of endophytic fungi communities in the roots of *Paphiopedilum barbigerum* from different habitats. Note: Left: Similarity clustering tree (UPGMA) based on species composition; Right: Stacked bar plot of taxonomic composition (grouped by sample). Colors represent species (see legend), and samples are grouped by site (HG, FQ, KY, YL, DE, KL, SL).

Unweighted pair-group method with arithmetic means (UPGMA) hierarchical clustering was performed and integrated with the relative abundance profiles of fungal taxa at the phylum level to illustrate evolutionary similarities and dissimilarities among microbial communities across environmental samples. Based on the Bray–Curtis distance matrix, the distribution of root-associated endophytic fungal communities at the genus level across the seven habitats revealed distinct compositional patterns. The HG site was predominantly characterized by *Epulorhiza* sp., and the FQ site by *Epulorhiza* sp. and *Fusarium solani*. The DE site was dominated by *F. solani*, *Verrucaria sublobulata*, and *Exophiala* sp., whereas the SL site harbored predominantly *Spizellomyces lactosolyticus*, *Exophiala brunnea*, and *Capronia* sp. At KL, the community was largely composed of *E. brunnea*, *Trichoglossum durandii*, and *Capronia* sp., while YL was characterized by *Exophiala* sp., *Paraboeremia putaminum*, and *E. brunnea*. The KY site was dominated by *Epulorhiza* sp. Pca-QS-0-1, *Clitopilus prunulus*, and *Hyalorbilia leguminacea*. The accompanying dendrogram showed that FQ and HG clustered together with high similarity, whereas KL, SL, YL, and DE formed another distinct cluster with greater compositional affinity (Figure 9).

### 3.8. LEfSe analysis of differentially abundant taxa across habitats

**Figure 10.**
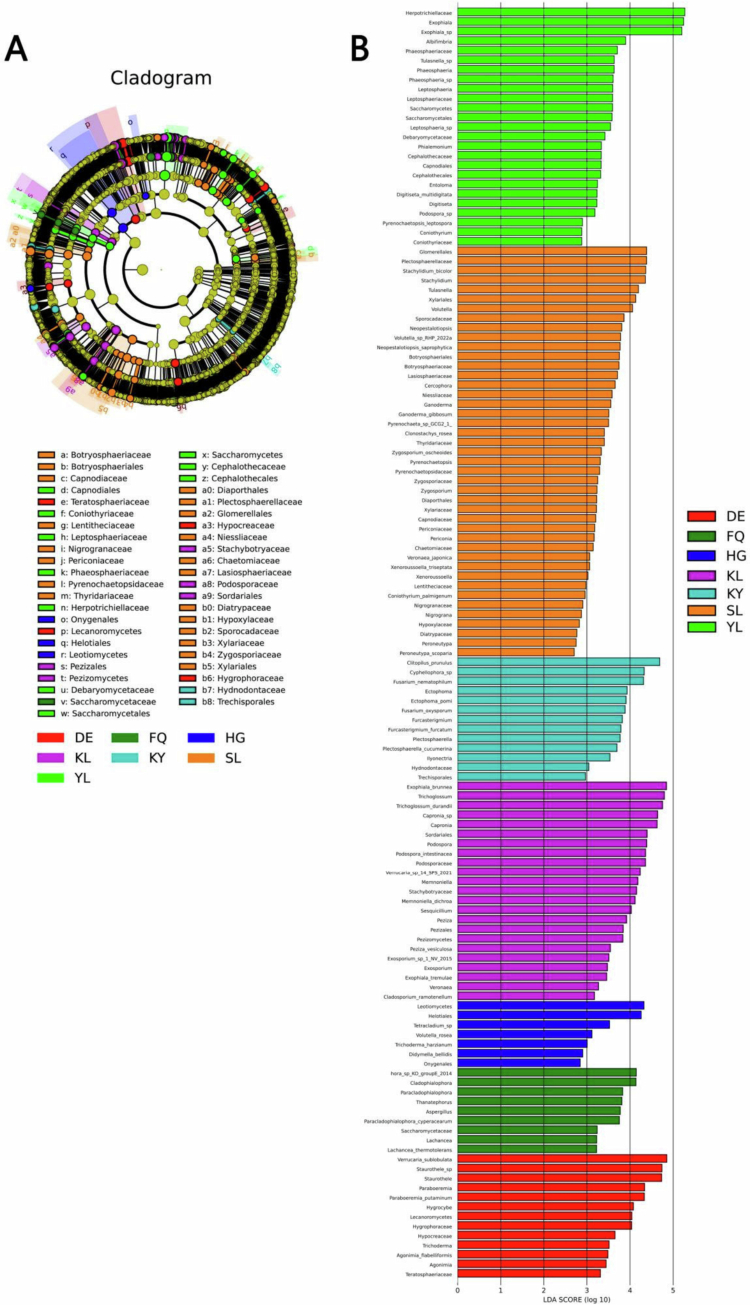
LEfSe (LDA Effect Size) cladogram and histogram depicting the endophytic fungi communities in the roots of *Paphiopedilum barbigerum* from different habitats.The results of the Kruskal–Wallis test indicated a statistically significant difference (*p* < 0.05). Note: Figure A: Circular cladogram (node colors represent sampling sites); Figure B: LEfSe LDA score plot (bar length indicates the discriminative power of taxa for sites, colors correspond to sites).

To identify statistically significant biomarkers and taxa that differentiate among groups, LEfSe analysis was performed on root samples from the seven habitats. The LDA results (LDA > 2, *P* < 0.05) revealed distinct sets of biomarkers for each site (Figure 10). The YL group harbored 25 biomarkers, among which Ascomycota-affiliated Eurotiomycetes, Chaetothyriales, Herpotrichiellaceae, *Exophiala*, and *Exophiala* sp. contributed most to group discrimination. The SL group contained 43 biomarkers, with Sordariomycetes, Glomerellales, Plectosphaerellaceae, *Stachylidium*, and *Stachylidium bicolor* (Ascomycota) as the strongest discriminators. The KY group had 13 biomarkers, primarily driven by Agaricomycetes, Agaricales, Entolomataceae, *Clitopilus*, and *Clitopilus prunulus* (Basidiomycota). The KL, HG, and FQ groups harbored 23, 7, and 9 biomarkers, respectively; for all three groups, the key contributors were Eurotiomycetes, Chaetothyriales, Herpotrichiellaceae, *Exophiala*, and *Exophiala brunnea* (Ascomycota). The DE group contained 13 biomarkers, with Eurotiomycetes, Verrucariales, Verrucariaceae, *Verrucaria*, and *Verrucaria sublobulata* (Ascomycota) contributing most to its differentiation. Collectively, these results demonstrate that habitat type shapes the composition of endophytic fungal taxa with significant discriminatory power among sites.

### 3.9. Identification of keystone taxa in co-occurrence networks across habitats

**Figure 11.**
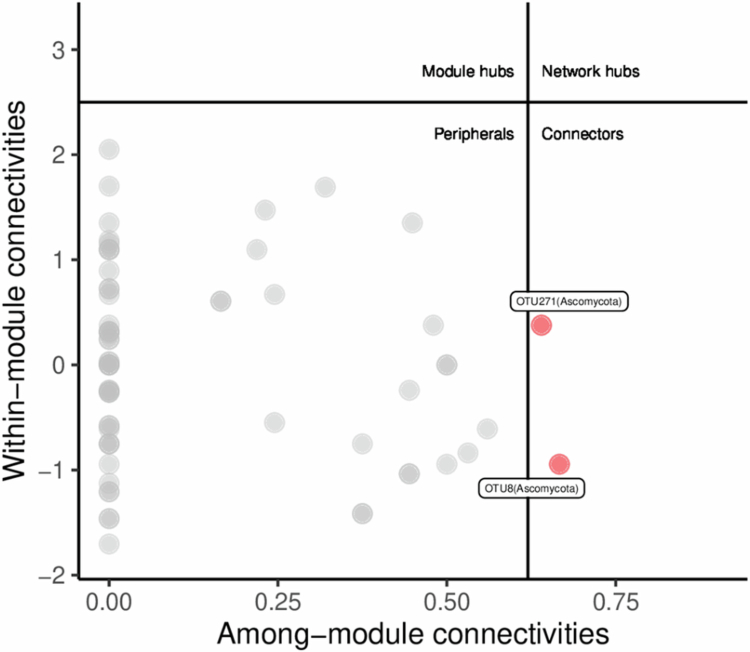
Analysis of endophytic fungi communities in the roots of *Paphiopedilum barbigerum* from different habitats Using the Zipi Index. Note: X-axis: Among-module connectivity; Y-axis: Within-module connectivity. Point color: Gray (non-hub nodes), Red (key hub nodes labeled as OTUs).

A co-occurrence network of endophytic fungi in *P. barbigerum* roots was constructed based on Spearman's rank correlations, and the ecological roles of individual OTUs were assessed using Zi-Pi analysis (Figure 11). The results showed that the vast majority of OTUs in the fungal community network were classified as peripherals (Zi < 2.5, Pi < 0.62). Only two connector nodes were identified (Zi < 2.5, Pi > 0.62)—OTU8 and OTU271, both belonging to Ascomycota—indicating that these taxa serve as keystone species maintaining connectivity among network modules. No module hubs or network hubs were detected, suggesting that the fungal community lacks absolutely dominant core taxa and relies primarily on a small number of connectors to coordinate interactions among modules.

## 4. Discussion

*Paphiopedilum barbigerum* belongs to a genus that lacks the pseudobulbs present in some other orchids, resulting in lower resilience and confining it largely to karst habitats with highly specialized ecological requirements.^32^ In this study, PacBio sequencing of root samples from seven distinct habitats yielded 792,178 clean reads and identified 2,310 fungal species across 8 phyla, 36 classes, 133 orders, 351 families, and 1,012 genera, with Ascomycota (52.90%–91.41%) and Basidiomycota (7.79%–54.33%) dominating the communities.

This study yielded three main findings. First, at the level of species diversity, habitat heterogeneity may be associated with community richness rather than evenness. Fungal species richness (Chao1 and ACE indices) in natural habitats within the reserve (SL, DE) was significantly higher than at the reintroduction site and in natural habitats outside the reserve (FQ, KY, HG). Second, at the level of community structure, habitat heterogeneity was significantly associated with fungal compositional divergence, while anthropogenic intervention may lead to community convergence. Beta diversity analysis and PERMANOVA testing (*R*² = 0.50, *P* = 0.001) confirmed that natural habitats formed distinct clusters, whereas HG and FQ—geographically separated yet both subject to human disturbance—exhibited highly similar community structures. Third, at the functional guild level, specific fungal taxa displayed consistent enrichment patterns across habitats, suggesting differentiated ecological roles. In natural habitats within the reserve, stress-tolerant Chaetothyriales and *Exophiala* were dominant; in contrast, at the reintroduction site and in natural habitats outside the reserve, the core OTUs comprised the symbiotic growth-promoting genus *Epulorhiza*, the potential biocontrol agent *Cladosporium*, and *Lachancea thermotolerans*, a yeast possibly involved in stress responses. Furthermore, the anomalously high abundance of *Fusarium* in disturbed habitats signals a potential ecological risk. Collectively, These findings suggest that the diversity of root-associated fungal communities of *P. barbigerum* is associated with habitat heterogeneity, and further indicate that the host may co-adapt with different functional guilds in response to heterogeneous environments.

### 4.1. Habitat heterogeneity drives fungal community structure and functional differentiation

Alpha diversity and LEfSe analyses (Figure 10) demonstrated that fungal richness and diversity in natural habitats within the reserve (SL, DE) were significantly higher than those at the reintroduction site and in natural habitats outside the reserve (FQ, KY, HG), and that community structure differed markedly among habitats, reflected the effects of habitat differences (Table 1). The study by^20^ revealed that the survival of *P. barbigerum* is strictly dependent on specific limestone cliff habitats with a rock exposure rate exceeding 70%, and that the key factors driving microbial community differentiation may be related to the habitat heterogeneity of the original sites.^20^ Tian et al.^18^ working on *Paphiopedilum* subgen. *Brachypetalum*, further demonstrated that even among physically similar native habitats, root-associated fungal community structure differed significantly across host species, which may reflect the association between habitat filtering and host-specific root microbiomes.^18^ Notably, despite their geographical separation, HG and FQ displayed highly similar fungal community structures (Figure 8). Anthropogenic disturbance, acting as a potent environmental filter, may be associated with habitat fragmentation and microhabitat homogenization, as well as with microbial community convergence at the reintroduction site and in disturbed habitats outside the reserve^33^—a pattern that may explain the reduced microbial diversity observed in these sites.

### 4.2. Adaptive symbiotic strategy of stress-tolerant fungal communities in natural habitats

In this study, the dominant taxa observed in the rock crevices of natural habitats within the reserve included those with stress-tolerant ecological strategies, including predominantly Chaetothyriales (23.13%–44.13%) and its representative genus *Exophiala* (12.58%–33.53%). Prior studies have shown that Chaetothyriales fungi generally possess a strong capacity to withstand nutrient limitation, drastic temperature and humidity fluctuations, and intense UV radiation.^34^ Suelgaray et al. ^35^ further indicated that Chaetothyriales fungi may possess a ‘pre-adaptation’ capability for the endophytic root environment.^35^ As a key genus within this order, *Exophiala* is a typical dematiaceous fungus that can extend the absorptive range of plant roots, improve the rhizosphere microenvironment, and enhance host water and nutrient uptake.^15^ ^,^ ^36^ We therefore propose that the enrichment of Chaetothyriales fungi, primarily Exophiala, in the roots of *P. barbigerum* within natural habitats in the reserve represents a critical adaptive symbiotic strategy that may help the host cope with the drought and nutrient stresses characteristic of karst habitats.

### 4.3. Dual nature of fungal communities in disturbed habitats: from symbiotic growth promotion to potential pathogenicity

In reintroduction habitats where soil substrate and moisture conditions are relatively improved but pathogen pressure or early-establishment stress may be present, the efficiency of rapid colonization and nutrient acquisition may covary with environmental conditions.Our study found that the reintroduction site and natural habitats outside the reserve (FQ, HG) were enriched in *Epulorhiza* (23.56%–44.79%) and *Fusarium* (28.76%–35.86%). *Epulorhiza*, a core symbiotic fungus of Orchidaceae, may enhance host stress tolerance through mycorrhizal symbiosis,^4^ ^,^ ^5^ ^,^ ^14^ ^,^ ^37-39^ whereas the pronounced enrichment of *Fusarium* suggests a potential risk of soil degradation or elevated pathogen pressure in disturbed environments.

*Fusarium* is a highly adaptive fungal genus that, under persistent environmental stress, can evolve multiple resistance mechanisms including target-site mutations and enhanced efflux pump activity. Numerous studies have shown that *Fusarium* includes many important plant pathogens,^40^ such as *F. solani*, *F. oxysporum*, *F. fujikuroi*, and *F. proliferatum*, which cause diseases in orchids and are responsible for major diseases in *Dendrobium* and other orchid genera.^41^ However, under favorable growth conditions, *F. proliferatum* ET1, *F. nematophilum*, *F. solani*, and *F. redolens* can colonize plants as asymptomatic endophytes.^4^ ^,^ ^42^ ^,^ ^43^ Certain *Fusarium* species can even promote seed germination and protocorm development in *Dendrobium officinale,* ^44^ ^,^ ^45^ although their growth-promoting effects may be weaker than those of obligate mycorrhizal fungi.^46^ ^,^ ^47^ These seemingly contradictory results may be related to the symbiotic-pathogenic duality of *Fusarium*, which is likely co-regulated by host physiological status and external environmental stress. In the disturbed habitats examined here, the high abundance of *Fusarium* may reflect a stressed host state. Collectively, these findings underscore the need for future studies to explore the dual potential and conditional triggers of endophytic and non-pathogenic *Fusarium* strains in pathogen suppression and the promotion of orchid germination and development.

### 4.4. Functional complementarity and synergistic adaptation of keystone symbiotic fungi in degraded habitats

Beta diversity analyses (PCoA, UPGMA) revealed that habitat heterogeneity was significantly associated with community structure divergence. Natural habitats such as SL and KL formed distinct clusters, whereas HG and FQ were associated with anthropogenic disturbance. This pattern was further reflected in the pronounced enrichment of cross-site core OTUs (*Epulorhiza* sp., *Cladosporium* sp., *Lachancea thermotolerans*) at FQ (46.30%) and HG (31.29%), suggesting that they may be associated with host adaptation in degraded habitats. *Epulorhiza* sp. (relative abundance up to 44.79%) is a well-documented keystone symbiotic fungi that effectively promotes seed germination and nutrient acquisition.^4^ ^,^ ^5^ ^,^ ^14^ ^,^ ^37-39^ *Cladosporium* sp., a common beneficial endophyte, can provide the host with biological protection through antagonism and competition^48^ ^,^ ^49^ and is consistently present across diverse orchid species.^50^ ^,^ ^51^ *L. thermotolerans*, although primarily reported in wine fermentation research,^52^ has recently been recognized for its plant growth-promoting potential and environmentally driven evolutionary background.^53^ ^,^ ^54^ Nevertheless, the symbiotic association between *P. barbigerum* and this fungus observed in this study, particularly its enrichment in degraded habitats, provides preliminary baseline data for exploring the potential roles of *Lachancea thermotolerans* in plant–microbe interactions.

We speculate that in degraded habitats, the challenges faced by *P. barbigerum* may shift from extreme drought stress to a combination of establishment competition, pathogen pressure, and nutrient acquisition efficiency. In this context, these three fungal taxa may co-exist and potentially perform complementary functions—such as biological protection, nutrient supply, and adaptive regulation—although this hypothesis requires further experimental validation.

Spearman network analysis revealed that the endophytic fungal community of *P. barbigerum* exhibits a decentralized and fragile network structure. The vast majority of OTUs were loosely connected peripheral nodes, with only a few key nodes (e.g., OTU8, OTU271) maintaining inter-module connections, and no core species were identified. This structure implies that *P. barbigerum* does not rely on a single core symbiotic fungus, but rather on a set of context-dependent key nodes. The loss of any such node may destabilize the symbiotic community and reduce the network's resistance to disturbance, thereby exacerbating the host's adaptive limitations in degraded habitats. Therefore, in conservation practices, attention should be given not only to the protection of the host plant itself, but also to the maintenance of fungal diversity associated with the original source habitats. Ex situ conservation and population reintroduction strategies should prioritize maintaining the population stability and functional integrity of these keystone symbiotic fungi, thereby helping to mitigate the extinction risk faced by this species due to its fragile symbiotic network.

### 4.5. Limitations

Finally, given the observational and correlational nature of this study, we cannot infer causality from the detected associations between habitat heterogeneity and fungal community assembly; our findings should therefore be interpreted as correlational rather than causal. Furthermore, several potentially important environmental variables—including soil pH, calcium ions (Ca²⁺), organic carbon, and water content—were not measured in this study and consequently could not be incorporated into our analytical models. Future studies that integrate comprehensive soil physicochemical data are needed to disentangle the relative contributions of habitat heterogeneity and edaphic factors in shaping root-associated fungal communities and host–fungus interactions.

## 5. Conclusion

This study provides the first demonstration that: (1) in natural habitats within the reserve, Chaetothyriales and *Exophiala* are strikingly dominant (relative abundance reaching 23.13%–44.13% and 12.58%–33.53%, respectively), suggesting they may assist the host in coping with karst drought stress by enhancing organic matter mineralization and water retention in rock crevices; (2) conservation strategies should prioritize the protection of natural habitats—the high-diversity fungal communities in Maolan Reserve native habitats (e.g., 1,012 species at SL) require strict limitation of anthropogenic disturbance to prevent the loss of functional guilds through habitat fragmentation; (3) the enrichment of *Epulorhiza* and *Cladosporium* at the reintroduction site points to the critical role of mycorrhizal symbiosis in seedling establishment, although community stability thresholds (core OTU abundance >30%) must be validated through long-term monitoring; and (4) the aberrant proliferation of *Fusarium solani* (28.76%) in disturbed areas may trigger functional imbalance in the fungal community, warranting metabolomics-based investigation into the regulatory thresholds governing its ‘growth-promotion–pathogenicity’ duality.

In summary, this study delineates the patterns of endophytic fungal community differentiation and identifies the core regulatory taxa across seven distinct habitats of *P. barbigerum*, providing baseline data for deciphering the symbiotic adaptation mechanisms of karst orchids and a scientific basis for mycorrhizal seedling cultivation and targeted application of core functional fungi in field reintroduction programs. It should be noted that while our high-throughput sequencing approach revealed correlations between community composition and habitat type, the specific functions of core fungal taxa (e.g., *Epulorhiza* sp.) and their interaction mechanisms with the host require further validation through isolation, culture, and symbiotic re-inoculation experiments. Future studies integrating multi-omics approaches—such as metabolomics, transcriptomics, and proteomics—are needed to deeply resolve the coupling mechanisms between fungal functional genes and host metabolic pathways, thereby providing a theoretical framework for precision intervention in habitat restoration and adaptive management of Orchidaceae plants.

## Data Availability

The datasets used or analyzed in the current study are available from the corresponding author upon reasonable request.

## References

[1] Gale S , Kumar P , Hinsley A , Cheuk M , Gao J , Liu H , Williams SJ . Quantifying the trade in wild-collected ornamental orchids in south China: diversity, volume and value gradients underscore the primacy of supply. Biol Conserv. 2019;238:14. doi: 10.1016/j.biocon.2019.108204.

[2] Long J , Qin F , Zhou J , Ran J , Qin L . Microenvironment characteristics of the endangered plant *paphiopedilum barbigerum* . J Guizhou Norm Univ (Nat Sci Ed). 2009;27:15–20. doi: 10.16614/j.cnki.issn1004-5570.2009.03.017.

[3] Shi J , Luo Y , Bernhardt P , Ran J , Liu Z , Zhou Q . Pollination by deceit in *paphiopedilum barbigerum* (Orchidaceae): a staminode exploits the innate colour preferences of hoverflies (Syrphidae). Plant Biol. 2009;11:17–28. doi: 10.1111/j.1438-8677.2008.00120.x. 19121110

[4] Tian F , Liao X , Wang L , Bai X , Yang Y , Luo Z , Yan F . Isolation and identification of beneficial orchid mycorrhizal fungi in *paphiopedilum barbigerum* (Orchidaceae). Plant Signal Behav. 2022;17:e2005882. doi: 10.1080/15592324.2021.2005882. PMC8920121 34913407

[5] Tian F , Wang J , Ding F , Wang L , Yang Y , Bai X , Tan C , Liao X . Comparative transcriptomics and proteomics analysis of the symbiotic germination of *paphiopedilum barbigerum* with *epulorhiza* sp. Fqxy019. Front Microbiol. 2024;15:1358137. doi: 10.3389/fmicb.2024.1358137. 38562471 PMC10982344

[6] Zhang J , Fang L , Zeng J , Li L , Wu K , Zeng S . Species and geographical distribution of *paphiopedilu* . Chin J Trop Crops. 2024;45:1572–1584. doi: 10.3969/j.issn.1000-2561.2024.08.006.

[7] Shade A , Jacques MA , Barrett M . Ecological patterns of seed microbiome diversity, transmission, and assembly. Curr Opin Microbiol. 2017;37:15–22. doi: 10.1016/j.mib.2017.03.010. 28437661

[8] Wang Z , Zhu Y , Jing R , Wu X , Li N , Liu H , Zhang X . High-throughput sequencing-based analysis of the composition and diversity of endophytic bacterial community in seeds of upland rice. Arch Microbiol. 2021;203:609–620. doi: 10.1007/s00203-020-02058-9. 32995980

[9] Li S , Sun M , Lei Y , Wang X , Liu H . Advances in the biological functions of seed endophytes and their mutual feedback with plants. Chin J Ecol. 2024;43:1959–1965. doi: 10.13292/j.1000-4890.202407.028.

[10] Wang M , Hu Y , Li H , Li J , Chen J , Lan S . New insights into the mycorrhizal fungi of orchidaceae plants. Guihaia. 2021;41:487–502. doi: 10.11931/guihaia.gxzw201907013.

[11] Xiong S , Li L , Yang H . Advances in research on enhancing drought resistance of plants by mycorrhizal fungi in orchidaceae. Mod Agric Sci Technol. 2024;102:92–97. doi: 10.3969/j.issn.1007-5739.2024.03.022.

[12] Li Y , Fang L , Chen H , Li L , Wu K , Zeng S . Advances in research on mycorrhizal fungi of *paphiopedilum* . Chin J Trop Crops. 2023;44:2157–2166. doi: 10.3969/j.issn.1000-2561.2023.11.003.

[13] Li Y , Chen H , Kong X , Yin Y , Li J , Wu K , Zeng S , Fang L . Excessive accumulation of auxin inhibits protocorm development during germination of *paphiopedilum spicerianum* . Plant Cell Rep. 2025;44:e03419. doi: 10.1007/s00299-024-03419-0. 39762613

[14] Wang W , Zou H , Dai Y , Lin J . Effects of *epulorhiza* on root morphology of *dendrobium officinale* and their symbiotic relationship. J Trop Subtrop Bot. 2020;28:124–130. doi: 10.11926/jtsb.4093.

[15] Wang J , Li T , Liu G , Smith J , Zhao Z . Unraveling the role of dark septate endophyte (DSE) colonizing maize (*zea mays*) under cadmium stress: physiological, cytological and genic aspects. Sci Rep. 2016;6:22028. doi: 10.1038/srep22028. 26911444 PMC4766571

[16] Pang Y , Ma D , Wang B , Cai Y , Wang J , Xiao S . Application of high-throughput sequencing technology in the study of plant endophytes. Chin J Biotechnol. 2024;40:3395–3406. doi: 10.13345/j.cjb.230873. 39467740

[17] Shamsudin NA , Seelan JSS , Gansau JA , Rusdi NA . A review: molecular identification of orchid mycorrhiza. Adv Hortic Sci. 2024;38:97–116. doi: 10.36253/ahsc-14952.

[18] Tian L , An M , Wu M , Liu F , Zhang Y . Habitat ecological characteristics and soil fungal community structure of *paphiopedilum* subgenus *brachypetalum* hallier (Orchidaceae) plants in southwest China. Plant Signal Behav. 2023;18:e2227365. doi: 10.1080/15592324.2023.2227365. PMC10308867 37377110

[19] Wang L , Wei L , Jiang Y , Pan D , Feng Y . Tissue culture and rapid propagation of *paphiopedilum barbigerum* . Plant Physiol Commun. 2010;46:1169–1170. doi: 10.13592/j.cnki.ppj.2010.11.019.

[20] Yu C , Bai P , Liu H , Chen Z , Mo J , Yao C , Fei S . Population structure and quantitative dynamics of *paphiopedilum barbigerum* in maolan karst forest. Subtrop Plant Sci. 2024;53:308–314. doi: 10.3969/j.issn.1009-7791.2024.04.003.

[21] Li H . Study on the characteristics of mycorrhizal fungal community and growth-promoting mechanism of paphiopedilum barbigerum in different habitats [Master’s thesis]. 2025. Guizhou University. doi: 10.27047/d.cnki.ggudu.2025.000210.

[22] Li Y , Hu J , Ruan Y , Wu Q , Yue Y , Li Z . Soil properties and rhizosphere microbes community structure reveal nitrogen uptake preferences and nitrogen use efficiency of two ecotypes of *paphiopedilum micranthum* . Agriculture. 2024;14:1909. doi: 10.3390/agriculture14111909.

[23] Jiang H , Chang J , Liao P , Lee Y . Breaking the hybrid myth of *paphiopedilum wenshanense:* double bifurcated divergence followed by adaptive introgression formed a morphological intermediate. Mol Ecol. 2025;34:e17613. doi: 10.1111/mec.17613. 39690874

[24] Edgar R . UPARSE: highly accurate OTU sequences from microbial amplicon reads. Nat Methods. 2013;10:996–998. doi: 10.1038/nmeth.2604. 23955772

[25] Edgar R . Search and clustering orders of magnitude faster than BLAST. Bioinformatics. 2010;26:2460–2461. doi: 10.1093/bioinformatics/btq461. 20709691

[26] Nilsson RH , Larsson KH , Taylor AFS , Bengtsson-Palme J , Jeppesen TS , Schigel D , Kennedy P , Picard K , Glöckner FO , Tedersoo L , et al. The UNITE database for molecular identification of fungi: handling dark taxa and parallel taxonomic classifications. Nucleic Acids Res. 2019;47:D259–D264. doi: 10.1093/nar/gky1022. 30371820 PMC6324048

[27] Luo J , Huang W , Zhang Q , Wu Y , Fang F , Cao J , Su Y . Distinct effects of hypochlorite types on the reduction of antibiotic resistance genes during waste activated sludge fermentation: insights of bacterial community, cellular activity, and genetic expression. J Hazard Mater. 2021;403:124010. doi: 10.1016/j.jhazmat.2020.124010. 33265039

[28] Xiong W , Song Y , Yang K , Gu Y , Wei Z , Kowalchuk GA , Xu Y , Jousset A , Shen Q , Geisen S . Rhizosphere protists are key determinants of plant health. Microbiome. 2020;8:9. doi: 10.1186/s40168-020-00799-9. 32127034 PMC7055055

[29] Segata N , Izard J , Waldron L , Gevers D , Miropolsky L , Garrett WS , Huttenhower C . Metagenomic biomarker discovery and explanation. Genome Biol. 2011;12:R60. doi: 10.1186/gb-2011-12-6-r60. 21702898 PMC3218848

[30] Chen C , Wang M , Zhu J , Tang Y , Zhang H , Zhao Q , Jing M , Xu X , Jiang J , Shen Z . Long-term effect of epigenetic modification in plant-microbe interactions: modification of DNA methylation induced by plant growth-promoting bacteria mediates promotion process. Microbiome. 2022;10:19. doi: 10.1186/s40168-022-01236-9. 35209943 PMC8876431

[31] Jiang Y , Sun B , Li H , Liu M , Chen L , Zhou S . Aggregate-related changes in network patterns of nematodes and ammonia oxidizers in an acidic soil. Soil Biol Biochem. 2015;88:101–109. doi: 10.1016/j.soilbio.2015.05.013.

[32] Shi J , Chen H , An M , Zhang Y , Ye C , Wu J . Status and conservation effectiveness analysis of wild *paphiopedilum* plant resources in guizhou province. Guihaia. 2022;42:1059–1066. doi: 10.11931/guihaia.gxzw202009038.

[33] Leng X , Zeng Y , Zhou J , Yang F , Ye J , Zhang J , Wu R . Analysis of habitat protection effectiveness of nature reserves in southwest China based on landscape fragmentation. Chin J Ecol. 2022;41:569–579. doi: 10.13292/j.1000-4890.202202.019.

[34] Lustosa B , Belmonte-Lopes R , De Hoog S , Costa F , Jacomel B , Dos Santos G , Razzolini E , Li Y , Xue R , Baura VA , et al. Genomics and ecology of epibryaceae, a psychrophilic family in chaetothyriales. IMA Fungus. 2025;16:170120. doi: 10.3897/imafungus.16.170120. PMC12750107 41476666

[35] Suelgaray F , Chiocchio V , Ciolfi F , Saparrat M . Are dark septate endophytes an ancestral ecological state in the evolutionary history of the order chaetothyriales? Arch Microbiol. 2023;205:6. doi: 10.1007/s00203-023-03401-6. 36607426

[36] Hu N . The mechanism of enhanced cadmium tolerance in host plants through the interaction between dark septate endophytic fungi (Exophiala pisciphila H93) and rhizosphere bacteria [Master’s thesis]. 2023. Yunnan University. doi: 10.27456/d.cnki.gyndu.2023.001653.

[37] Li B , Tang M , Tang K , Zhao L , Guo S . Screening for differentially expressed genes in *anoectochilus roxburghii* (Orchidaceae) during symbiosis with the mycorrhizal fungus *epulorhiza* sp. Sci China Life Sci. 2012;55:164–171. doi: 10.1007/s11427-012-4284-0. 22415688

[38] Swangmaneecharern P , Serivichyaswat P , Nontachaiyapoom S . Promoting effect of orchid mycorrhizal fungi *epulorhiza* isolates on seed germination of *dendrobium* orchids. Sci Hortic. 2012;148:55–58. doi: 10.1016/j.scienta.2012.09.013.

[39] Jin H , Xu Z , Chen J , Han S , Ge S , Luo Y . Interaction between tissue-cultured seedlings of *dendrobium officinale* and mycorrhizal fungi during co-culture. Chin J Plant Ecol. 2009;33:433–441. doi: 10.3773/j.issn.1005-264x.2009.03.002.

[40] Naqvi S , Farhan M , Ahmad M , Kiran R , Shahbaz M , Abbas A , Hakim F , Shabbir M , Tan YS , Sathiya Seelan JS . Fungicide resistance in *fusarium* species: exploring environmental impacts and sustainable management strategies. Arch Microbiol. 2025;207:24. doi: 10.1007/s00203-024-04219-6. 39792175

[41] Tsavkelova E , Kolomeitseva G . *Fusarium*-orchid interactions under greenhouse conditions. S Afr J Bot. 2022;146:889–896. doi: 10.1016/j.sajb.2022.03.038.

[42] Latiffah Z , Hayati MN , Baharuddin S , Maziah Z . Identification and pathogenicity of *fusarium* species associated with root rot. Asian J Plant Pathol. 2009;3:14–21. doi: 10.3923/ajppaj.2009.14.21.

[43] Srivastava S , Kadooka C , Uchida J . *Fusarium* species as pathogen on orchids. Microbiol Res. 2018;207:188–195. doi: 10.1016/j.micres.2017.12.002. 29458853

[44] Shah S , Paudel MR , Thapa BB , Sharma H , Kashyap AK , Rekadwad BN , Pant B . Extract from endophytic *fusarium* isolates stimulates seed germination of the host and protocorm development of non-host orchids. Commun Integr Biol. 2025;18:2439798. doi: 10.1080/19420889.2024.2439798. 39703375 PMC11654709

[45] Chen X , Dong H , Hu K , Sun Z , Chen J , Guo S . Diversity and antimicrobial and plant-growth-promoting activities of endophytic fungi in *dendrobium loddigesii* rolfe. J Plant Growth Regul. 2010;29:328–337. doi: 10.1007/s00344-010-9139-y.

[46] Vujanovic V , St-Arnaud M , Barabé D , Thibeault G . Viability testing of orchid seed and the promotion of colouration and germination. Ann Bot. 2000;86:79–86. doi: 10.1006/anbo.2000.1162.

[47] Swett C , Uchida J . Characterization of *fusarium* diseases on commercially grown orchids in hawaii. Plant Pathol. 2015;64:648–654. doi: 10.1111/ppa.12290.

[48] Islam M . Current status and future prospects of *cladosporium* sp., a biocontrol agent for sustainable plant protection. Biocontrol Sci. 2022;27:185–191. doi: 10.4265/bio.27.185. 36567114

[49] Zhu M , Zhang W , Duan X , Yan S , Cai Y , Gong S , Fahad S , Qiu Z . Biocontrol potential of *cladosporium sphaerospermum* against the wheat powdery mildew fungus blumeria graminis f. Sp. tritici. Plant Dis. 2024;108:2983–2988. doi: 10.1094/pdis-02-24-0433-sc. 38654537

[50] Chen J , Hu K , Hou X , Guo S . Endophytic fungi assemblages from 10 *dendrobium* medicinal plants (Orchidaceae). World J Microbiol & Biotechnol. 2011;27:1009–1016. doi: 10.1007/s11274-010-0544-y.

[51] Tan X , Chen X , Wang C , Jin X , Cui J , Chen J , Guo S , Zhao L . Isolation and identification of endophytic fungi in roots of nine *holcoglossum* plants (Orchidaceae) collected from yunnan, guangxi, and hainan provinces of China. Curr Microbiol. 2012;64:140–147. doi: 10.1007/s00284-011-0045-8. 22057921

[52] Vicente J , Navascues E , Calderon F , Santos A , Marquina D , Benito S . An integrative view of the role of *lachancea thermotolerans* in wine technology. Foods. 2021;10:2878. doi: 10.3390/foods10112878. 34829158 PMC8625220

[53] Ramos-Garza J , Aguirre-Noyola J , Bustamante-Brito R , Zelaya-Molina L , Maldonado-Hernández J , Morales-Estrada A , Resendiz-Venado Z , Palacios-Olvera J , Angeles-Gallegos T , Terreros-Moysen P , et al. Mycobiota of Mexican maize landraces with auxin-producing yeasts that improve plant growth and root development. Plants. 2023;12:1328. doi: 10.3390/plants12061328. 36987016 PMC10058334

[54] Hranilovic A , Bely M , Masneuf-Pomarede I , Jiranek V , Albertin W . The evolution of *lachancea thermotolerans* is driven by geographical determination, anthropisation and flux between different ecosystems. PLoS One. 2017;12:e0184652. doi: 10.1371/journal.pone.0184652. 28910346 PMC5599012

